# *Burkholderia cepacia complex* Cervical Osteomyelitis in an Intravenous Drug User

**DOI:** 10.1155/2018/7638639

**Published:** 2018-09-09

**Authors:** Sui Kwong Li, William B. Messer

**Affiliations:** ^1^Division of Infectious Diseases, Oregon Health & Science University, 3181 SW Sam Jackson Park Road, Mail Code L457, Portland, Oregon 97239, USA; ^2^Department of Microbiology and Immunology, Oregon Health & Science University, 3181 SW Sam Jackson Park Road, Mail Code L220, Portland, Oregon 97239, USA

## Abstract

Gram-negative vertebral osteomyelitis infections are increasing due to rising intravenous drug use but overall remain uncommon. Here, we present a case of *Burkholderia cepacia complex* cervical osteomyelitis in an intravenous drug user. *Burkholderia cepaciacomplex* vertebral osteomyelitis has been infrequently described in the literature thus far with varied antibiotic treatment regimens. A 68-year-old male presented to the emergency department with neck pain after minor trauma. He endorsed active intravenous heroin and methamphetamine use. CT and MRI imaging of the cervical spine revealed destructive changes of C5-C6 vertebral bodies consistent with osteomyelitis. Neurological exam was stable and vital signs were within normal limits; so, antibiotics were held, and he was admitted for diagnostic evaluation. Five sets of blood cultures were drawn on admission and were ultimately negative. He subsequently underwent C5-C6 corpectomy, C4-C7 anterior fusion, and C3-T1 posterior fusion with allograft placement. Deep operative tissue cultures grew *Burkholderia cepacia complex*. He was treated with 6 weeks of intravenous ceftazidime followed by indefinite oral minocycline due to hardware placement. *Burkholderia cepacia complex* should be considered among pathogenic etiologies of pyogenic vertebral osteomyelitis, particularly among patients with intravenous drug use. Ceftazidime monotherapy was an effective treatment in this particular case.

## 1. Introduction

Pyogenic vertebral osteomyelitis is a highly morbid and debilitating disease process typically acquired via hematogenous seeding of bacteria in the highly vascular vertebral bone marrow.

Though microbiologic etiology typically consists of a monomicrobial Gram-positive organism [1], the incidence of polymicrobial and Gram-negative bacterial infections is increasing, largely due to the ongoing intravenous opioid abuse epidemic [2–4].

*Burkholderia cepacia complex* are closely related aerobic Gram-negative bacilli that are well described in infections among immunocompromised and cystic fibrosis patients [5]; however, their role in infection and pathogenicity has been less well described among immunocompetent adults. There have been scarce case reports of *Burkholderia cepacia complex* vertebral osteomyelitis in the literature [6–8], and antibiotic therapy has been historically varied. Here, we report a case of *Burkholderia cepacia complex* cervical osteomyelitis in an intravenous drug user who was successfully treated with ceftazidime monotherapy in the setting of spinal stabilization hardware placement.

## 2. Case Presentation

A 68-year-old male with a past medical history of schizophrenia and polysubstance abuse presented to the emergency department with a chief complaint of neck pain following minor trauma after sustaining a mechanical fall complicated by a television set then falling on him, striking him on the back of his neck. He denied loss of consciousness, paresthesias, or focal weakness but reported an increasing number of falls for the past two years. He endorsed daily intravenous heroin and methamphetamine use with social alcohol use and having previously quit smoking twenty years ago.

Vital signs were temperature 37.3°C, blood pressure 141/108 mmHg, heart rate 105 beats per minute, respiratory rate 24 breaths per minute, and oxygen saturation 97% on room air. Physical examination revealed a thin, cachectic African American male. He was noted to be without obvious signs of trauma to the head or neck. Cardiac examination revealed sinus tachycardia with no obvious murmurs, rubs, or gallops. Pulmonary examination was clear to auscultation in all lung fields. Abdominal examination was soft without evidence of distention or tenderness to palpation in all four quadrants. Skin examination showed no evidence of rashes, erythema, or trauma. Neurological examination was significant for cervical spinal tenderness to palpation but without evidence of step-off or gross deformity. Strength was noted to be diffusely diminished (4/5 throughout bilateral upper and lower extremities) without hyperreflexia or pathologic reflexes (negative Babinski and Hoffman tests). Anal wink was present. Sensation was preserved throughout. Laboratory results demonstrated white blood cell count 5540/mm^3^ with normal differential, serum creatinine 1.6 mg/dL (baseline 0.6 mg/dL), C-reactive protein (CRP) 25.2 mg/L, and erythrocyte sedimentation rate (ESR) 48 mm/hr, and urine drug screen was positive for amphetamines and opiates. HIV testing was negative. Hepatitis C testing revealed a positive antibody test but a negative viral load, suggestive of cleared prior infection. He denied prior treatment of Hepatitis C.

Computed tomography (CT) and magnetic resonance imaging (MRI) of the cervical spine revealed lytic destructive changes involving the C5 and C6 vertebral bodies, concerning for osteomyelitis. Due to clinical stability, antibiotics were held and he was admitted to the hospital for planned CT-guided aspiration. Five sets of blood cultures drawn over two consecutive days while off antibiotics were negative. Transthoracic echocardiography revealed a 3 × 4 mm echodensity attached to the noncoronary cusp of the aortic valve that was not independently mobile and without associated aortic regurgitation. In addition, there was a 3 × 5 mm echodensity attached to the anterior leaflet of the mitral valve that was not independently mobile and without associated mitral regurgitation.

During his hospitalization, the patient remained afebrile and hemodynamically stable but began to report tingling in his right thumb and index finger and decreased dexterity in terms of handwriting and grip strength, resulting in dropping objects. Due to new neurologic symptoms, the patient went to the operating room on hospital day #3 and underwent C5-C6 corpectomy, C4-C7 anterior fusion with allograft, and C3-T1 posterior fusion with allograft. Intraoperative findings demonstrated soft bone from the inferior portion of C4 to the superior portion of C7 with granulation tissue noted at the posterior longitudinal ligaments. There was no obvious evidence of tissue necrosis or purulent material. Postoperative vancomycin and piperacillin/tazobactam were started. Deep operative cultures subsequently grew *Burkholderia cepacia complex*, identified by matrix-assisted laser desorption/ionization time-of-flight mass spectrometry (MALDI-TOF MS). Susceptibilities determined by the Kirby–Bauer method revealed susceptibility to ceftazidime, trimethoprim/sulfamethoxazole, and minocycline, with intermediate resistance to levofloxacin.

His antibiotics were narrowed to intravenous ceftazidime 2 g every 8 hours. He was evaluated by the Improving Addiction Care Team (IMPACT) and started on medication-assisted treatment for his opioid use disorder with Suboxone while being an inpatient. He was discharged to a skilled nursing facility where he completed 6 weeks of ceftazidime. At the end of his intravenous antibiotic therapy, his CRP was noted to be 0.7 mg/L, and his ESR was 39 mm/hr. He was prescribed indefinite minocycline 100 mg by mouth twice daily due to vertebral hardware placement. He subsequently missed his follow-up clinical and opiate counseling appointments and was ultimately lost to follow-up.

## 3. Discussion

*Burkholderia cepacia complex* vertebral osteomyelitis is an infrequently described clinical syndrome in the literature though it has previously been associated with intravenous drug use [6]. Treatment can be challenging due to intrinsic resistance to multiple drug classes via efflux pumps, antibiotic degradation and/or modifying enzymes, and altered membrane function [9]. In addition, the placement of spinal stabilization hardware increased the likelihood of planktonic- and biofilm-embedded organisms. Despite this, based on his clinical improvement and improved CRP and ESR over his course of intravenous antibiotic therapy, we believe we were successfully able to treat this infection with ceftazidime monotherapy. Unfortunately, we are so far unable to assess the outcome of long-term minocycline suppression as the patient was lost to follow-up.

We were initially concerned about the possibility of native-valve infective endocarditis given the echocardiographic findings; however, in review of the echocardiogram with the reading cardiologist, these findings were felt to be most consistent with prior rather than active endocarditis. This interpretation was supported by his clinical stability while under observation prior to his operation and his serial negative blood cultures drawn while the patient was off antibiotics. Due to clinical stability and need for indefinite suppressive antibiotics, we did not utilize combination antimicrobial therapy.

The microbiologic diagnosis of *Burkholderia cepacia complex* can be challenging depending on which modality is being utilized to identify the organism. In this case, we utilized MALDI-TOF MS; other automated systems have reported a misidentification rate of up to 31% [10]. In addition, clinical outcomes may vary as a byproduct of which bacterium subspecies within the *complex* is pathogenic. *Burkholderia cenocepacia* has been associated with worse clinical outcomes compared to *Burkholderia multivorans* in patients with cystic fibrosis [11, 12]; however, our microbiology lab does not routinely perform subspeciation of *Burkholderia cepacia complex*.

The importance of surgical intervention in this case cannot be understated, both in terms of infectious source control and pathogen identification. In light of the ongoing opiate-abuse epidemic, clinicians will need to maintain a higher index of suspicion for *Burkholderia cepacia complex* as a causative pathogen for pyogenic vertebral osteomyelitis.
